# Periodontal Disease Monitoring by Raman Spectroscopy of Phosphates: New Insights into Pyrophosphate Activity

**DOI:** 10.3390/diagnostics14010066

**Published:** 2023-12-27

**Authors:** Eduard Gatin, Stefan Marian Iordache, Dina Ilinca Gatin, Pal Nagy, Ana-Maria Iordache, Catalin Luculescu

**Affiliations:** 1 Faculty of Medicine, University of Medicine and Pharmacy “Carol Davila”, Blv. Eroii Sanitari 8, Sector 5, 050474 Bucharest, Romania; 2 Faculty of Physics, DMSPA Department, University of Bucharest, Atomistilor Str. 405, 077125 Magurele, Romania; 3 Optospintronics Department, National Institute for Research and Development for Optoelectronics—INOE 2000, Atomistilor Str. 409, 077125 Magurele, Romania; stefan.iordache@inoe.ro (S.M.I.); ana.iordache@inoe.ro (A.-M.I.); 4 Faculty of Dentistry, University of Medicine “Carol Davila”, Calea Plevnei 17-23, Sector 5, 0110221 Bucharest, Romania; dinailinca@yahoo.com; 5 Faculty of Dentistry, Periodontology Department, Semmelweiss University, 1085 Budapest, Hungary; kardpali@gmail.com; 6 CETAL Department, National Institute for Laser, Plasma and Radiation Physics, Atomistilor Str. 409, 077125 Magurele, Romania

**Keywords:** Raman spectroscopy, octacalcium phosphate (OCP), hydroxyapatite (HAP), pyrophosphate (PPi), periodontal disease

## Abstract

(1) Background: The intent of this survey was to investigate the quality of the alveolar bone by revealing the different phases for calcified tissues independent of the medical history of the patient in relation to periodontal disease by means of Raman spectroscopy and then to correlate the results by suggesting a possible mechanism for the medical impairment; (2) Methods: The investigation was mainly based on Raman spectroscopy that was performed in vivo during surgery for the selected group of patients. The targeted peaks for the Raman spectra were according to the reference compounds (e.g., calcium phosphates, other phosphates); (3) Results: The variation in the intensity of the spectrum correlated to the specific bone constituents’ concentrations highlights the bone quality, while some compounds (such as pyrophosphate, PPi) are strongly related to the patient’s medical status, and they provide information regarding a physiological process that occurred in the calcified tissues. Moreover, bone sample fluorescence is related to the collagen (Col) content, enabling a complete evaluation of bone quality, revealing the importance of collagen matrix acting as a load-bearing element for Calcium phosphate (CaP) deposition during the complex bone mineralization process; (4) Conclusions: We highlight that Raman spectroscopy can be considered a viable investigative method for in vivo and rapid bone quality valuation through oral health monitoring.

## 1. Introduction

Nowadays, there is a focused interest for periodontal disease because it depicts a significant haleness provocation. The genesis of human research makes efforts in periodontology to understand the mechanism involved in the development of calcified tissues (implicating calcium phosphate compounds, phosphates and different enzymes), with the final goal to completely rebuild the lost tissues with a structure and functionality similar to the original one, which were lost due to the disease progression or to prevent or limit the disease progression [1,2,3,4,5]. In order to achieve this final goal, the bone tissue evaluation and investigation are necessary. An easy-to-use, fast and comprehensive method is Raman spectroscopy. It is rapidly arising as a bright device for biomedical investigations and clinical diagnosis, such as the detection of early-stage cancer, and it has been validated in countless ex vivo studies [4,5,6]. Recently, Raman spectroscopy has been applied to the investigation of bone quality in bioimplants [7]. Timchenko and his team performed a comparative analysis of the surfaces of mineralized and demineralized bone bioimplants. The bones were sourced from cadaveric and in vivo (resected) samples. The two-dimensional analysis showed that carbonate concentration in the resected bone was higher than in cadaveric bone, which induced higher resistance to demineralization for the resected bone. There are few spectroscopic studies on bones [8,9], and the literature is even scarce for spectroscopic data on alveolar bone.

The periodontal regeneration process is defined as the formation of new cementum, alveolar bone and a functional periodontal ligament on a previously diseased root surface. Our other studies have focused on connecting the Raman spectra of the mineral and organic phases of the bone with histology/histomorphometry [10] and establishing a connection between periodontal disease and bone phenotype with Raman spectra of calcium phosphate compounds [3]. In this study, we focus only on spectral characterization of the alveolar bone; its investigation led us to suggest a possible mechanism regarding amorphous Ca-phosphate (ACP) compound starting with octacalcium phosphate (OCP) final transformation into hydroxyapatite (HA).

Some studies were performed recently regarding the quality of dental enamel [11] and using human teeth for evaluation for calcium phosphate (CaP) deposition during bone mineralization that starts with the aggregation of Posner’s clusters Ca_9_(PO_4_)_6_ into amorphous Ca-phosphate (ACP). An important role is played by the alkaline phosphatase (ALP), which releases phosphate ions for mineralization [12]. Octacalcium phosphate (OCP), a bone substitute material, is considered a precursor of the biological bone apatite. The two-layered structure of OCP contains the apatitic and hydrated layers and is intensively involved in ion-exchange surface reactions, which results in OCP hydrolysis to hydroxyapatite (HA) and adsorption of ions or molecular groups presented in the environment as motion species ((HPO_4_)^2−^, Ca^2+^, OH^1−^). The composition of the solution and environmental structure (collagen matrix) affects the degree and rate of OCP hydrolysis, its surface reactivity and further in vitro and in vivo properties [13].

In the present study, we attempted to carry out an in vivo characterization of mineralization processes by Raman spectroscopy in patients with or without periodontitis. By doing so, we try to analyze the mineralization products, with an emphasis on pyrophosphate, and to provide some insight into the related mechanism [14,15]. Our attempt is another step toward developing and bringing the Raman spectroscopy method to the clinics as the end or final users.

## 2. Materials and Methods

**Figure 1:**
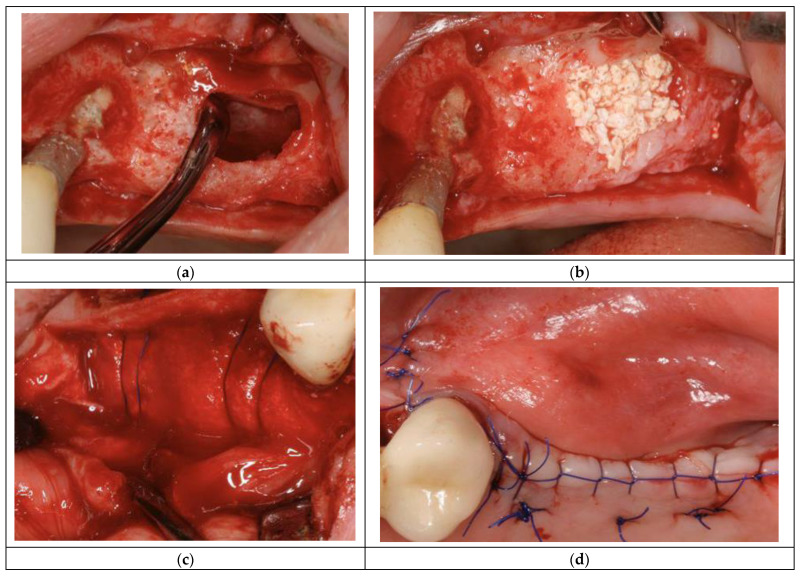
Maxillary sinus floor elevation (patient #3): lateral bony window with the ‘trap-door’ technique (**a**), sinus filled with inorganic bovine bone mineral (**b**), lateral window covered by resorbable collagen membrane (**c**), wound closure (**d**).

For the present study, a group selection of ten (10) patients was made. All patients were under medical surveillance and had a very clear clinical status reported either as healthy, previous periodontal or periodontal. The bone pieces harvested were part of standard clinical care, and details regarding the group of patients are listed in the table below (Table 1). The patients come to the clinic for maxillary/mandibular dentures. During clinical examination and subsequent surgical interventions, bone samples were removed with a micro drill (from a physio dispenser), and their clinical status was determined (permanent dentures can only be fixed on healthy bone; the patients who exhibited disease symptoms underwent treatment before surgical interventions)—Figure 1. According to a medical valuation of the patients and their medical status achieved, a color code was labeled to each one as follows: green (• healthy), blue (• previous periodontal) and red (• periodontal). For every patient, a surgical procedure was indicated on an edentulous alveolar ridge site. As a starting point for our study, the Raman spectroscopy technique was involved primarily in patients’ oral sites of interest for in vivo evaluation [5]. The ex vivo experimental assembly is presented elsewhere (Figure 1a in [10]). 

The maxillary sinus floor elevation oral surgery is a relatively common procedure usually used in conjunction with implantology for patients with periodontal disease. The principal steps of the abovementioned procedure are presented in Figure 1.

Raman spectroscopy was performed after the first step (Figure 1a) using a replaceable and autoclavable optical fiber head. The Raman measurement was performed for about one second. The next step in the surgical procedure was to fill the sinus with a commercial bovine bone material (Figure 1b). The lateral window was covered with a resorbable collagen membrane (Figure 1c), and the wound was closed by a suitable number of sutures (Figure 1d).

### 2.1. In Vivo Measurements

For in vivo investigation of the patients, the Raman probe was fitted with a special ‘cap’ tailored to be compatible with steam autoclave sterilization according to the medical standard protocol. The special cap contains a slot for inserting the Raman probe equipped with a spacer sleeve that fits and holds the Raman probe in a fixed position (vertical).

Before evaluation, the targeted area of the jawbone was prepared with blood suction, washed with saline and kept dry as much as possible by blood aspiration. During data acquisition, other electromagnetic wave sources were avoided (no light, lamps off) in order to have far less possible fluorescence contamination from the krypton lamp. The Raman probe was fixed almost in a perpendicular position on the jawbone surface (interest site) chosen for examination (Figure 1a in [10]). For each patient, three Raman spectra were recorded during the in vivo evaluation [3,5,16,17].

All patients signed an informed consent. The biopsy and Raman investigation protocol were approved by the Semmelweis University Regional and Institutional Committee of Science and Research Ethics (SE TUKEB No. 234/2015, No. 141/2020). 

The starting point for our investigation is based mainly on OCP and additionally on HAP (amorphous hydroxyapatite air-dried and air-damped), HAP crystalline (from Sigma–Aldrich, St. Louis, MO, USA), two bone substitutes based on biological HAP (Cerabone^®^, Botiss Zossen, Berlin, Germany) and Bio–Oss (Geistlich Korea Biomaterials MBH, Princeton, NJ, USA); all were shaped as pellets for easy handling. The first three pure calcium phosphates (OCP, HAP amorphous air-dried and HAP amorphous air-damped) were synthesized at the Frantsevich Institute—Kyiv, Ukraine, as described and included their characteristics in a previous study [3].

### 2.2. Characterization Methods

Raman spectra were acquired by using a BTR111—785 RAMAN spectrometer device (λ = 785 nm, output power p = 300 mW and spectral resolution as fine as 4 cm^−1^) in the shift range of 300–1800 cm^−1^ for all samples’ investigation, both in vivo and ex vivo evaluation. The integration time was 1000 ms, and laser power was fitted for 10% of maximum output power (300 mW). Regarding the Maximum Permissible Exposure (MPE) for bone tissue, there is no clear limitation for the skin (0.50 mW/cm^2^) according to the American National Standard Institute (ANSI). During ‘in vivo’ measurements employed in our study, the laser irradiation level (ablation process) was about ≈7 mW/mm^2^. The Raman spectrometer was calibrated with a Si (100) spectroscopic standard sample, before and after data recording in both cases of in vivo and ex vivo measurements. Experimental data were recorded under the same geometrical conditions during bone sample evaluation, in three points corresponding to in vivo and ex vivo, in order to avoid local heating, and with dark acquisition before each data recording. Data processing was performed by using Origin Pro v2017 software (Origin Lab, Northampton, MA, USA).

Selected values for Raman peak intensities were obtained after baseline correction and unit normalization was applied to raw data (dark subtracted, not affected by noise, summarized peak to peak). Differences in peak intensity on raw spectra reflect the differences in the quantities of the chemical components for each investigated specimen. Sensitive qualitative/quantitative information may be obtained according to the Raman spectra shape, such as fluorescence by using raw data (no flat line subtraction and without smoothing) [5,15,17,18].

To summarize, the methodology consisted in recording the Raman spectra (using a replaceable and sterilizable optical fiber head) for different types of bones (healthy, periodontal or healed) before the patient underwent surgery. Then, the Raman spectra were compared with the level of mineralization of the bone (immature, mature and type-B carbonate) in order to propose a calcification process mechanism.

## 3. Results and Discussion

Raman investigation highlights the peaks (Raman shift) for the main bone (cortical or cancellous type) components (chemical groups and elements) in order to evaluate differences between bone tissue for the investigated patients (healthy, previous periodontal or periodontal). The assignment of the main Raman peaks has been detailed elsewhere [3,5] in table form. Briefly, we observed high-intensity peaks corresponding to extensive mineral immature bone (at 955–960 cm^−1^, 955 cm^−1^, 957 cm^−1^) [15], to mineral mature bone (peaks at 960–965 cm^−1^, 963 cm^−1^) [15,19], peaks corresponding to symmetric P••O stretch modes of PO_3_^2−^ moieties, ν_3_ PO_3_ and of P–O–P bridging (at 1023 cm^−1^ and 1027 cm^−1^—attributed to PPi (P_2_O_7_^4−^), inorganic pyrophosphate) [20,21,22]. Mineral bone B-type carbonate HAP presented high-intensity peaks at 1070 cm^−1^ and 1076 cm^−1^ [15], while ν_3_PO_4_^3−^ showed peaks at 430–450 cm^−1^ (with shoulder) and 1048 cm^−1^ [14,15,19]. The selected Raman bands (shifts) of bone tissue have been established to be significant to our study and for the future tracking of chemical compounds and evaluation/monitoring of the patients.

**Figure 2:**
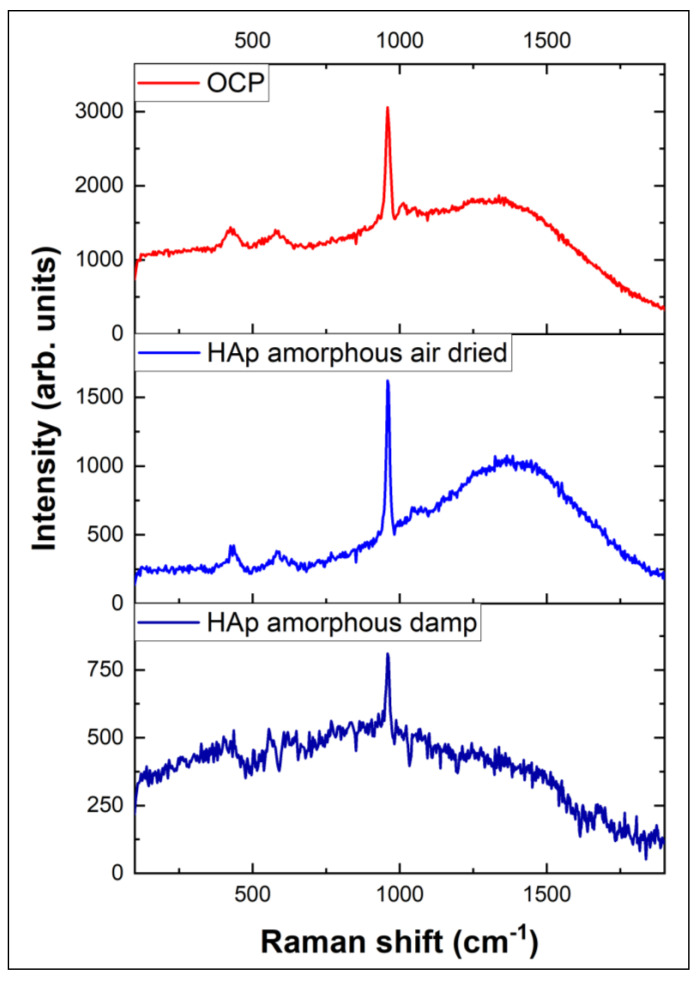
Raman spectra for basic calcium phosphate compounds.

**Figure 3:**
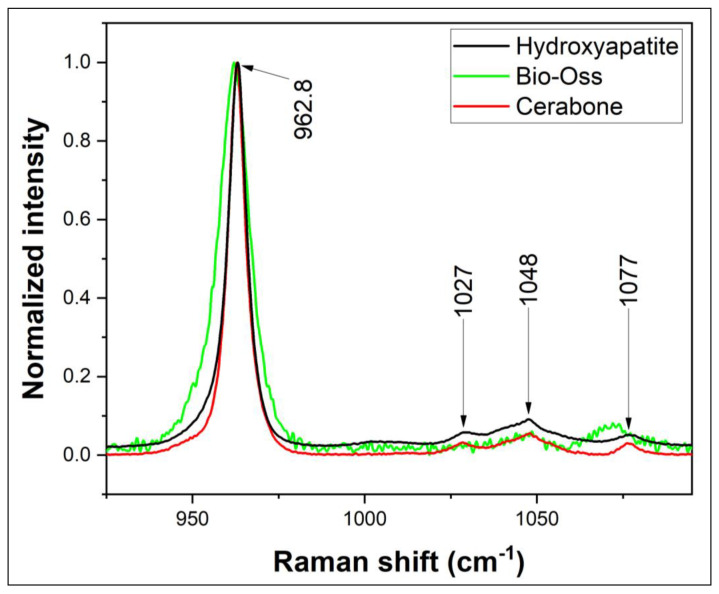
Raman spectra for hydroxyapatite and bone substitutes.

As was mentioned before, the starting point and discussion will be focused on OCP, because it is considered a natural precursor of HAP in the process of natural bone mineralization. Therefore, it is believed that, compared with other calcium phosphates, it has the most pronounced bioactivity properties, including osteoinductiveness [3,23]. For this reason, in our study, it is considered to be the stage from where the mineralization process can be developed and metabolism can be involved. Raman spectra obtained for the reference calcium phosphate compounds (OCP, HAP—air-dried and air-damped amorphous phase), respectively, for HAP crystalline phase and bone substitutes (Cerabone and Bio–Oss) are depicted in Figure 2 and Figure 3. Some of the Raman bands (shift) that are observed in Figure 2 and Figure 3 overlap the assignment of the main Raman peaks. 

The presence of PPi was noticed for the OCP compound, HAP amorphous phases and for bone substitutes Cerabone/Bio–Oss Geistlich. For the HAP crystalline phase acquired from Sigma Aldrich, it was absent. A stronger PPi peak was noticed for OCP and HAP air-damped (Raman signal weaker), as OCP is considered the starting point of the transition phase to HAP. A lower intensity value was observed for Cerabone and Bio–Oss bone substitutes. For those phosphate compounds, even being in a high-crystalline phase, soft traces of PPi were found, a fact that confirms their biological origin. 

In a previous work [5], we established the patients’ status based on Ca/P ratio, clinical evaluation and Raman bands statistics. The same group of patients was used for the present investigation. After a careful evaluation of in vivo Raman investigation results, ‘some regulation’ was noticed regarding the rates of pyrophosphate peak intensities reported to those of HAP peak intensities. 

**Figure 4:**
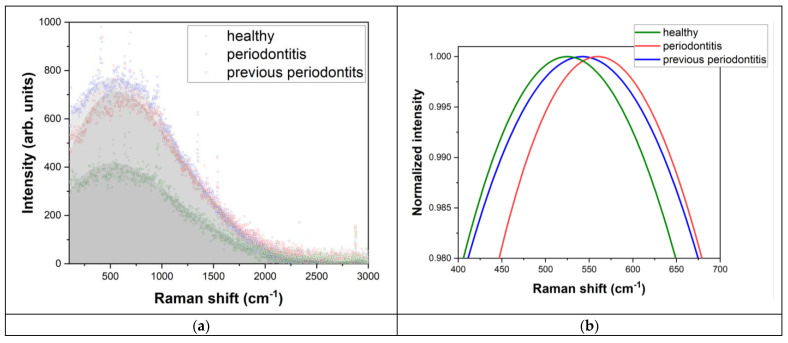
Luminescence corresponding to selected patients according to medical status (healthy, previous periodontitis and periodontitis). (**a**) Raw data for Raman spectra; (**b**) Normalized values of Col for Raman shift related to reference value 550 cm^−1^.

The investigation for the present study was completed with collagen (Col) evaluation related to the second-order luminescence band at ~500–900 cm^−1^ (817–845 nm), as shown in Figure 4. Additionally, the following rates related to (Col) were introduced, as described by I_Col_/I_PO4_ (Table 2) and I_Col_/I_Pyro_ (Table 3), where I is the intensity of Raman peaks in arbitrary units for specific elements. The Raman results presentation and discussion are systematized according to the patient’s medical status after the clinical evaluation. 

The patient’s status information depicted in Table 1 after correlation with the Raman investigation results from Table 2 and Table 3, with the additional information from pyrophosphate levels, could make the prediction of periodontal clinical status much easier.

The Raman results presentation and discussion are systematized according to the patient’s medical status, emphasizing a possible mechanism for the periodontal disease. We have the following categories:

(I) Clinical status—Periodontal healthy (patients: #2, #6 and #9)

For this category, for the rate, I_col_/I_PO4_ values are centered on 1.00 (oscillating in the interval (0.95 ÷ 1.12). In the case of the I_Col_/I_Piyro_ ratio, the situation is similar and oscillates in the interval (1.00 ÷ 1.03).

(II) Clinical status—Previous periodontitis (patients: #1, #3, #4, #5 and #8)

For this category, values obtained have the same trend, and I_Col_/I_PO4_ is oscillating in the interval (0.93 ÷ 1.08). Regarding the rate I_Col_/I_Piyro_, the obtained values belong to the interval 1.00 ÷ 1.19. 

(III) Clinical status—Periodontitis (patients: #7 and #10)

**Figure 5:**
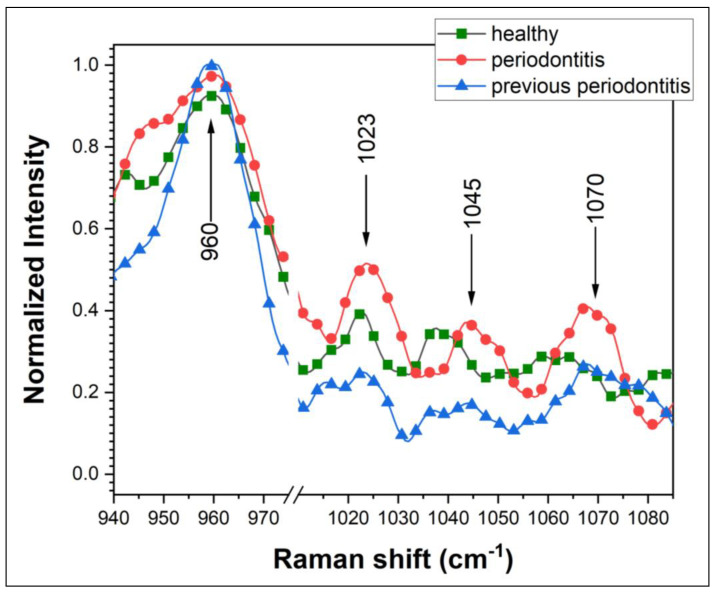
Raman spectra, representative for the patient’s medical status (healthy, previous periodontitis and periodontitis).

For this category, a total imbalance was noticed between the components ratio, and they are significantly different than the other two categories. The ratio for I_Col_/I_PO4_ was the highest and, respectively, the lowest of all the values calculated for the patients (the two values obtained were 1.26 and 0.89), while the ratio corresponding to the I_Col_/I_Piyro_ showed the lowest values of all (0.84 and 0.96). A typical difference between the categories described above is depicted in Figure 5.

These results confirm previously obtained results [5]. An important remark is that the rates reported for Col, and taking into account Col luminescence, are more sensitive and accurate. A possible mechanism for periodontal disease can emerge by taking into account Col and Pyro behavior applied on phase transition for calcium phosphates starting with OCP.

Regarding the ratio to (PO_4_ and HPO_4_^2−^) corresponding to 959.6 cm^−1^ Raman shift, the obtained values are almost the same with small variations ±0.10 around the 1.00 value. A higher imbalance can be observed for periodontal patients (#7 and #10), which confirms their medical status. The Raman shift corresponds to an extensive mineral immature bone (Table 1). As a suggested mechanism, for the phase transition mechanism to mature bone (Raman shift 962.6 cm^−1^), the pyrophosphate is involved. According to the ratio to Pyro (Raman shift 1023/1027 cm^−1^), the level is higher, which may be interpreted as a shortage of this compound for periodontal patients. For the bone tissue, there is P_i_ ((H_2_PO_4_)^1−^, (HPO_4_)^2−^ and (PO_4_)^3−^) as a mixture of ions including PP_i_ ((P_2_O_7_)^4−^) under the physiological pH (homeostatic). In some conditions, the balance can be broken and the mineralization process is affected. 

### Proposed Phase Transition Mechanism Starting with OCP Considered Precursor for HAP

Some of the current studies are using different calcium phosphate compounds very close to HAP as a Posner molecules cluster model (Ca_9_(PO_4_)_6_) or CDHA (Calcium Deficient Hydroxyapatite, Ca_9_(HPO_4_)(PO_4_)_5_OH) as a transition to HAP [12,24,25]. We suggest as a starting point a ‘crude’ calcium phosphate compound as OCP and more implication of metabolism and tissue elements to the final transition for HAP. 

As was mentioned before, for the bone tissue there is a mixture of ions including Ca^2+^ and (OH)^1−^ (water elimination by hydrolysis) as well. Some of the motion species (P_i_ and PP_i_) are the result of ATP (adenosine triphosphate) metabolism. A very important chemical reaction occurs with two of the motion species being involved:

(1) $${\left(P_{2}O_{7}\right)}^{4-}+H_{2}O+energy\overset{{Mg}^{2+}}{\to }2{\left({HPO}_{4}\right)}^{2-}$$

The obtained (HPO_4_)^2−^ (Equation (1)) is involved in water generation by deprotonation (not released, for HAP transition) and in a higher concentration is typical for immature bone (amorphous phase). 

For the transition OCP → HAP, we presume intrusion of Ca^2+^ and (OH)^1−^ to the OCP elementary cell network (by chelation and disruption of the water layer bound on the crystal surface) that is achieved with the mechanical action (W, Work) of cementoblast/osteoblast cells supported by proteins (PV—Phosvitin, PPP—Phosvitin phosphopeptides) and enzymatic activity of TNAP, ANK and NPP1 during homeostasis in the extracellular space [25,26,27,28,29,30].

**Figure 6:**
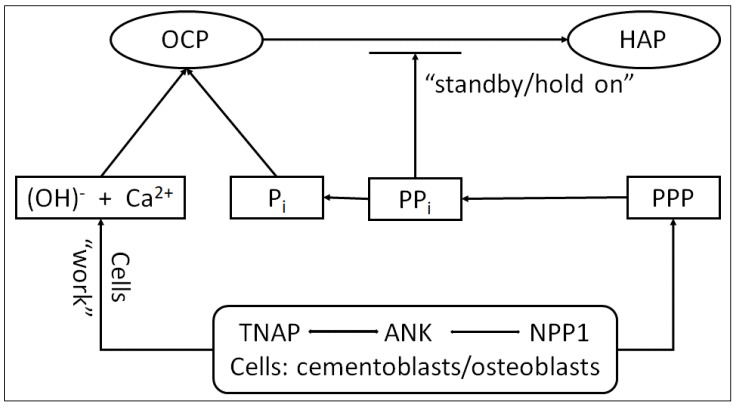
The suggested mechanism for OCP transition to HAP and possible explanation for periodontal disease.

Inorganic phosphate (Pi) is a component of mineral hydroxyapatite (HAP), while pyrophosphate (PPi) is a potent inhibitor of HAP crystal precipitation and growth, according to Foster et al. [27]. The tissue enzyme nonspecific alkaline phosphatase (TNAP) hydrolyzes PPi to release ionic Pi, creating conducive conditions for mineralization. The local PPi level is increased by the functions of the progressive ankylosis protein (ANK) and ectonucleotide pyrophosphatase phosphodiesterase 1 (NPP1), which act to preserve the mineralization process on ‘standby’ (Figure 6 and [24]). 

Many studies are focused on defining the regulatory role of PPi in tooth root cementum development and demonstrated that PPi acts as a basic regulator for tooth root acellular cementum development, a determinant key defining the hard–soft interface between the cementum and PDL (Periodontal Ligaments). Dysregulation of PPi resulting from loss of any of the central PPi controlling factors explored here had profound consequences on the development of acellular extrinsic fiber cementum (AEFC), a tissue essential to tooth attachment and function. To wit, loss of TNAP caused severe underdevelopment or even absence of acellular cementum. Because these three factors, TNAP, ANK and NPP1, primarily adjust extracellular PPi, then support PPi as the key mechanistic factor, this prompts us to propose that PPi regulates acellular cementum in a molecular ‘rheostat’ fashion, i.e., acellular cementum thickness relates inversely to PPi production [24,25], thus resulting in the importance of a quick and precise method for monitoring, such as Raman spectroscopy. According to the obtained results, the trend was fully confirmed by the obtained Raman peaks and ratios depicted in Table 2 and Table 3.

Based on other suggested models [24,27,28,31], we propose a model for the hypothesized role of PPi in the phase transition of OCP to HAP that is essential in acellular cementum formation (Figure 6). The periodontal region is very rich in ALP (alkaline phosphatase) activity (reducing local PPi) and thus a permissive milieu action for cementum formation on the root surface. In the course of normal development, cementoblasts modulate PPi to curb apposition (by increasing PPi via ANK and NPP1) to maintain AEFC (acellular extrinsic fiber cementum) as a thin tissue on the root surface. When one of these PPi factors is removed from the equation, apposition cannot be fully regulated and cementoblasts attempt to compensate by increasing the expression of its counterpart PPi regulator [32,33,34,35]. With directly controlling cementum mineral apposition, these studies suggest that PPi influences ECM (extracellular matrix) protein composition [36,37,38,39]. In the circumstances of rapid cementogenesis, cementoblasts are increasing the expression of OPN (osteopontin) and DMP1 (dentin matrix phosphoprotein). The increase in OPN, a negative regain of HAP crystal growth, may be an additional cementoblast mechanism employed to limit the extent of cementum apposition [40,41,42].

## 4. Conclusions

The present study aimed to promote the Raman spectroscopy as a new simple, quick, noninvasive and independent method of investigation for in vivo application in oral surgery regarding bone evaluation in relation to periodontal disease (even disease mechanism) or bone healing status in oral reconstructive surgery [43,44,45,46,47]. 

Mainly, the investigation was based on tracing the two compounds (PO_4_^3−^/HPO_4_^2−^ and PPi) related and reported to Col. The rates obtained from Table 2 and Table 3 confirmed the proposed mechanism and are sustained by the medical status of the patients. The key role is held by PPi, but in close relation with other elements as proteins and enzymes. The big advantage is that PPi is Raman-sensitive, compared to enzymes that can be monitored only by their activity (TNAP, not isolated). 

The proposed model for a phase transition is based on similar models but starts with OCP and emphasizes some physicochemical processes such as chelation and ‘Work’ to be involved. 

An important limitation of our study is that of the small number of patients who were involved in the investigation, compared with other studies (~600,000 patients) [35,48]. Another limitation is that some enzymes (TNAP) are not isolated, and information can be obtained just by monitoring the activity. 

To highlight: # Raman spectroscopy accuracy is capable of differentiating the patients’ status as healthy/periodontitis recovered/periodontitis and to use PPi as periodontitis marker in the future; # The proposed model can be a new start for metabolism implication in bone tissue recovery; # The study can be a call for a larger number of patients in order to validate the proposed method.

## Figures and Tables

**Table 1:** Listed patients involved in the study, including details regarding their clinical status and bone type. For each case, color-coding was used: (• healthy), (• previous periodontal), (• currently periodontal).

Patient Number	Age (Years) and Gender	Clinical Status	Bone Phenotype/Bone Type
#1	58M	Previously periodontal	Thick/more cortical
#2	70M	Healthy	Thick/more cortical
#3	64M	Previously periodontal	Thin/more cortical
#4	50F	Previously periodontal	Thick/more cortical
#5	70M	Previously periodontal	Thin/more cortical
#6	35M	Healthy	Thin/more cortical
#7	62F	Currently periodontal	Thin/more cancellous
#8	37F	Previously periodontal	Thin/cortical–cancellous
#9	45F	Healthy on the lower jaw, but previously periodontal on the upper jaw	Thin/more cortical
#10	43M	Currently periodontal	Thick/more cortical

**Table 2:** Ratio of collagen reported to ν_1_PO_4_ intensities. The color codes in the table are related to healthy (green), with previous periodontitis (blue) and present periodontitis (red) clinical conditions of the patients.

Patient	PL Maximum (cm^−1^)	Intensity (arb.u.)	Intensity PO_4_ (@959.6 cm^−1^) (arb.u.)	I_Col_/I_PO4_
#1	726.65	1550.18	1439.33	1.07
#2	673.55	748.43	672.66	1.11
#3	641.91	330.80	334.75	0.98
#4	692.39	847.90	783	1.08
#5	686.13	376.71	358.41	1.05
#6	574.47	765.16	742.25	1.03
#7	635.55	812.51	641.16	1.26
#8	567.98	402.07	483.75	0.93
#9	574.47	510.37	523.75	0.97
#10	515.57	371.83	413.91	0.89

**Table 3:** Ratio of collagen photoluminescence (col) intensity versus inorganic pyrophosphate (Pyro) intensities. The color codes in the table are related to healthy (green), with previous periodontitis (blue) and present periodontitis (red) clinical conditions of the patients.

Patient	PL Maximum (cm^−1^)	Intensity (arb.u.)	Intensity Pyro (@1023/1027 cm^−1^) (arb.u.)	I_Col_/I_Pyro_
#1	726.65	1550.13	1433	1.08
#2	673.55	748.43	724	1.03
#3	641.91	330.80	293	1.12
#4	692.39	847.90	709	1.19
#5	686.13	376.71	340	1.10
#6	574.47	765.16	752	1.01
#7	635.55	812.51	962	0.84
#8	567.98	402.07	402	1.00
#9	574.47	510.37	502	1.01
#10	515.57	371.83	386	0.96

## Data Availability

Data are available upon request to the corresponding authors in a reasonable time frame.
